# Inhibition of Store-Operated Calcium Entry Attenuates MPP^+^-Induced Oxidative Stress via Preservation of Mitochondrial Function in PC12 Cells: Involvement of Homer1a

**DOI:** 10.1371/journal.pone.0083638

**Published:** 2013-12-17

**Authors:** Xia Li, Wei Chen, Lei Zhang, Wen-bo Liu, Zhou Fei

**Affiliations:** 1 Department of Neurosurgery, Xijing Institute of Clinical Neuroscience, Xijing Hospital, Fourth Military Medical University, Xi’an, Shaanxi, China; 2 Department of Neurosurgery, The First Affiliated Hospital of Xi’an Jiaotong University, Xi’an, Shaanxi, China; Cornell University, United States of America

## Abstract

The process of store-operated calcium entry (SOCE), whereby the release of intracellular Ca^2+^ from endoplasmic reticulum (ER) activates Ca^2+^ influx channels in the plasma membrane, has been demonstrated to impact a diverse range of cell functions. In the present study, we investigated the potential protective effect of SOCE inhibition against 1-methyl-4-phenylpyridinium (MPP^+^) injury by using pharmacological antagonists or specific small interfering RNA (siRNA) in PC12 cells. The results showed that both antagonists (15 μM MRS-1845 and 50 μM ML-9) and stromal interacting molecule-1 (STIM1) targeted siRNA (Si-STIM1) significantly increased cell viability, decreased apoptotic cell death and reduced intracellular reactive oxygen species (ROS) production and lipid peroxidation in MPP^+^ injured PC12 cells. SOCE inhibition also prevented MPP^+^ induced mitochondrial dysfunction and activation of mitochondrial related apoptotic factors, while had no effect on mitochondrial biogenesis. Moreover, inhibition of SOCE by antagonists and siRNA increased the expression levels of Homer1a mRNA and protein, and knockdown of Homer1a expression by specific siRNA partly reversed the protective effects induced by SOCE inhibition in PC12 cells. All these results indicated that SOCE inhibition protected PC12 cells against MPP^+^ insult through upregulation of Homer1a expression, and SOCE might be an ideal target for investigating therapeutic strategy against neuronal injury in PD patients.

## Introduction

 Parkinson disease (PD), first reported by James Parkinson in 1817, is the second most common neurodegenerative disorder and affects about 2% of the population over the age of 60 [1]. It is characterized by clinical symptoms including rigidity, bradykinesea, resting tremor and postural instability, and by neuropathological features involving loss of dopaminergic (DA) neurons and formation of cytoplasmic lewy bodies in the substantia nigra and substantia innominate [2]. The mechanism that underlies the progressive degeneration of DA neurons is not clear, and current therapies for PD are inadequate because of failure to provide constant levels of dopamine replacement and modify the underlying neuronal degeneration [3]. However, abnormal calcium homeostasis is believed to be a major component in the pathogenesis of nigral cell death and might be an ideal target for identification of potential therapeutic strategies for PD patients [4,5].

Cytosolic calcium acts as a ubiquitous second messenger and Ca^2+^-mediated cellular stress has long been thought to be important in neurodegenerative disease including PD [6]. The unusual reliance of DA neurons on calcium in autonomous pacemaking suggests that stress of storage organelles (such as mitochondria and endoplasmic reticulum) created by calcium homeostasis dysfunction could be responsible for their selective vulnerability, rather than simply a late-stage consequence [7]. Calcium signals are generated by both the release of stored calcium from the endoplasmic reticulum (ER) and the influx of extracellular calcium across the plasma membrane. The entry of extracellular calcium generally results from depletion of intracellular stores in a process referred to as capacitative calcium entry or store-operated calcium entry (SOCE), which not only ensures optimal refilling of the endoplasmic reticulum, but also leads to a prolonged increase in cytosolic Ca^2+^ [8]. A previous study showed that platelet SOC channel is a crucial mediator of ischemic cardiovascular and cerebrovascular events [9], and a spontaneous decreased SOC channels activity was also demonstrated in familial Alzheimer’s disease-linked presenilin-1 mutation M146V transfected human neuroblastoma SK-N-SH cells [10]. However, there are no studies to date of the effect of SOCE inhibition on MPP^+^ induced neuronal injury. In this study, we sought to investigate the influence of SOCE inhibition by using pharmacological antagonists or siRNA-induced gene silencing in an in vitro PD model involving cultured PC12 cells treated with 1-methyl-4-phenylpyridinium (MPP^+^). MPP^+^ was added into the culture medium at the concentration of 500 μM for 24 h to induce cell death [11,12]. We also determined the effects of SOCE inhibition on the expressions of Homer 1a mRNA and protein after MPP^+^ insult, and investigated the relationship between SOCE inhibition induced neuroprotection and its modulatory effects on Homer1a. 

## Materials and Methods

### Materials

Dulbecco’s modified Eagle’s medium (DMEM) and fetal bovine serum were purchased from Gibco (Gaithersburg, MD, USA). ML-9, MRS1845 (MRS), Fura-2 AM, EGTA, MPP^+^, Triton X-100 and Hoechst 33342 were purchased from Sigma (St. Louis, MO, USA). The terminal deoxynucleotidyl transferase-mediated dUTP nick end labeling (TUNEL) staining assay kit was purchased from Promega (Madison, WI, USA) and the bicinchoninic acid (BCA) protein assay kit was obtained from Pierce (Rockford, IL, USA). The fluorescent dye rhodamine 123 (Rh123) and dichlorofluorescein diacetate (DCF-DA) were obtained from Pierce (Rockford, IL, USA). 

### Cell culture and treatment

PC12 (adrenal gland; pheochromocytoma) cells were obtained from the Institute of Biochemistry and Cell Biology, SIBS, CAS. The cells were grown in DMEM plus 10% fetal bovine serum and 1% antibiotics (penicillin/streptomycin). The medium was changed every 2-3 days. In all experiments, the cells were pretreated with ML-9 (50 μM) or MRS (15 μM) for 30 min before the addition of MPP^+^ at the final concentration 500 μM for an additional 24 h. All experiments were repeated five times for each treatment condition in each experiment.

### Cell viability assay

Cell viability assay was performed by using The Cell Proliferation Reagent WST-1 following the manufacture’s protocol (Roche, Basel, CH). After treatment, 10 μl cell proliferation reagent, WST-1, was added to each well and incubated for 4 hours at 37 °C and 5% CO_2_. Then, 100 μl/well culture medium and 10 μL cell proliferation reagent, WST-1, as used in the experiment, is added into one well (e.g. 100 μL culture medium plus 10 μl cell proliferation reagent, WST-1). This background control (absorbance of culture medium plus WST-1 in the absence of cells) is used as a blank position for the ELISA reader. Shake thoroughly for 1 minute on a shaker and measure the absorbance of the samples against a background control as blank using a microplate (ELISA) reader.

### siRNA transfection

 PC12 cells were seeded into 6-well plates the day before transfection. The sequence of STIM1 siRNA was CAGCTTTGAGGCCGTCCGCAA. The sequence of Homer1a was GCATGCAGTTACTGTATCTTC. The negative control siRNA was AATTCTCCGAACGTGTCACGT, which should not knock down any known proteins. The above siRNA molecules were chemically synthesized by Genechem Company (Shanghai, China), and the transfection was performed by using transfection reagent Lipofectamine 2000 according to the manufacturer’s protocol (Invitrogen, Sacramento, CA, USA). The siRNA transfection efficiency was approximately 80%, estimated by Block-iT Alexa Fluor Red Fluorescent Oligo (Invitrogen). 

### Calcium imaging

Intracellular Ca^2+^ concentration was measured using the calcium indicator Fura-2-AM as previously described [13]. Cultured PC12 cells grown on glass slides were loaded with 5 μM Fura-2 AM in control solution for 45 min before MPP^+^ treatment at room temperature. The coverslips were then gently washed to remove the extracellular dye and were placed in a cuvette filled with pre-warmed control solution or Ca^2+^-free solution. Control solution contained: 120 mM NaCl, 4.75 mM KCl, 1.2 mM MgCl_2_, 20 mM HEPES, 1.8 mM CaCl_2_, 10 mM glucose, 1.17 KH_2_PO_4_ and 0.05 % BSA. In Ca^2+^-free solution CaCl_2_ was omitted and 200 μM EGTA was added. Excitation wavelength was alternated between 340 nm and 380 nm and the emitted fluorescence was collected at 510 nm. Results are presented as the ratio of 340/380 fluorescence.

### TUNEL staining

Apoptotic cell death was detected by the TUNEL assay, a method to observe DNA strand breaks in nuclei. Briefly, PC12 cells were seeded on 1.5 cm glass slides at a density of 3 × 10^5^ cells/cm^2^. Twenty-four hours after MPP^+^ treatment, cells were fixed by immersing slides in freshly prepared 4 % methanol-free formaldehyde solution in PBS for 20 min at room temperature and permeabilized with 0.2 % Triton X-100 for 5 min. Cells were labeled with fluorescein TUNEL reagent mixture for 60 min at 37°C according to the manufacturer's suggested protocol (Promega, Madison, WI, USA). After that, slides were examined by fluorescence microscopy and the number of TUNEL-positive (apoptotic) cells was counted. Hoechst 33342 (10 μg/ml) was used to stain nucleus. Apoptotic rate was represented as a percentage of the total number of cells.

### Measurement of caspase-3 activity

 The activity of caspase-3 was measured using the colorimetric assay kit according to the manufacturer’s instructions (Cell Signaling Technology, MA, USA). Briefly, after being harvested and lysed 10^6^ cells were mixed with 32 μL of assay buffer and 2 μL of 10 mM Ac-DEVD-pNA substrate. Absorbance at 405 nm was measured after incubation at 37 °C for 4 h. Absorbance of each sample was determined by subtraction of the mean absorbance of the blank and corrected by the protein concentration of the cell lysate. The results were described as relative activity to that of control group.

### Measurement of intracellular ROS production

Briefly, PC12 cells were incubated with DCF-DA (10 μM) for 1 h at 37 °C in the dark, and then resuspended in PBS. Intracellular ROS production was detected using the fluorescence intensity of the oxidant-sensitive probe 2, 7-dichlorodihydrofluorescein diacetate (H2DCFDA) in an Olympus BX60 microscope and fluorescence was read using an excitation wavelength of 480 nm and an emission wavelength of 530 nm.

### Measurement of lipid peroxidation

Malonyldialdehyde (MDA) and 4-hydroxynonenal (4-HNE), two index of lipid peroxidation, were determined by using assay kits from Cell Biolabs and strictly following the manufacturer’s instruction (Cell Biolabs, San Diego, CA, USA). The absorbance of the samples was measured by a microplate (ELISA) reader.

### Measurement of mitochondrial membrane potential (MMP)

 MMP was measured using the fluorescent dye Rh123 as reported previously [13]. MMP depolarization resulted in the loss of Rh123 from the mitochondria and a decrease in intracellular fluorescence. Rh 123 was added to cultured neurons to achieve a final concentration of 10 μM for 30 min at 37°C after the cells had been treated and washed with PBS. The fluorescence was observed by using an Olympus BX60 microscope with the appropriate fluorescence filters (excitation wavelength of 480 nm and emission wavelength of 530 nm). The MMP level was calculated from the fluorescence value using a calibration curve, and it is expresses as % of control.

### Measurement of intracellular ATP

The intracellular ATP level was measured by using ApoSENSOR Cell Viability Assay Kit obtained from Biovision and strictly following the manufacturer’s protocol (Biovision, San Francisco, CA, USA). ATP concentration of each treatment was calculated as a percentage of control.

### Measurement of permeability transition pore (MPTP)

 MPTP was measured by a fluorescence assay kit (Genmed, Shanghai, China) according to the method previously reported [14]. Briefly, PC12 cells were collected by centrifugation at 300Ⅹ*g* for 5 min, then 500 μL preheated cleaning solution (Reagent A) and equal amounts of staining working solution which contained staining solution (Reagent B) and neutralization solution were added to the cell suspension, respectively. Next they were mixed gently and incubated in the dark at 37 °C for 20 min. At last the samples were resuspended in Reagent A again and detected by using fluorescence spectrophotometer. 

### Quantification of cytochrome c release

 Cytochrome c release into the cytoplasm was assessed after subcellular fraction preparation. PC12 cells in 6-well plates were washed with ice-cold PBS for three times and lysed with a lysis buffer (50 mM Tris–HCl pH 7.5, 150 mM NaCl, 1 mM EDTA, 0.1 % SDS, 0.2 % deoxycholic acid and 1:100 protease inhibitor cocktail). The cell lysate was centrifuged for 10 minutes at 750 *g* at 4 °C, and the pellets containing the nuclei and unbroken cells were discarded. The supernatant was then centrifuged at 15 000 *g* for 15 minutes. The resulting supernatant was removed and used as the cytosolic fraction. The pellet fraction containing mitochondria was further incubated with PBS containing 0.5% Triton X-100 for 10 minutes at 4 °C. After centrifugation at 16 000 *g* for 10 minutes, the supernatant was collected as mitochondrial fraction. The protein content in each fraction was determined using a BCA protein assay kit and the levels of cytochrome c in cytosolic and mitochondrial fractions were measured using the Quantikine M Rat/Mouse Cytochrome C Immunoassay kit obtained from R&D Systems. Data were expressed as ng/mg protein.

### Real-time RT-PCR

Total RNA was prepared from PC12 cells as previously described [15]. The expression level of D-loop, ATP8, PGC-1, NRF-1, TFAM and Homer 1a mRNA was determined by real-time reverse transcriptase-polymerase chain reaction (RT-PCR), and the primer sets are shown in Table 1. The condition of amplification was: 5 min at 94 °C; 35 cycles of 45 s at 94 °C, 1 min at 56 °C, 1 min at 72 °C; followed by 10 min at 72 °C. The relative expression value was normalized to the expression value of GAPDH.

**Table 1:** Primers sequences used in real-time PCR.

**Gene**	**Forward sequences**	**Reverse sequences**
**D-loop**	5’-AGGCATCTGGTTCTTACTTC-3’	5’-TGACGGCTATGTTGAGGA-3’
**ATP8**	5’-CTTCCCAAACCTTTCCTG-3’	5’-GGTAATGAAAGAGGCAAATAGA-3’
**PGC-1**	5’-GTGCAGCCAAGACTCTGTATGG-3’	5’-GTCCAGGTCATTCACATCAAGTTC-3’
**NRF-1**	5’-GAGTGACCCAAACCGAACA-3’	5’-GGAGTTGAGTATGTCCGAGT-3’
**TFAM**	5’-GGTGTATGAAGCGGATTT-3’	5’-CTTTCTTCTTTAGGCGTTT-3’
**Homer1a**	5’-CGGAATTCTATCTTCAGCACTC-3’	5’-CGGAATTCAATGCATTCTGAGC-3’
**GAPDH**	5’-ATGTATCCGTTGTGGATCTGAC-3’	5’-CCTGCTTCACCACCTTCTTG-3’

### Determination of mitochondrial respiratory chain complex I activity

 Mitochondria were purified by Percoll density gradient centrifugation in extraction buffer (50 mM Tris HCl, pH 7.5, 500 mM NaCl, 0.03 % reduced Triton X-100, 1 mM EDTA, 1 mM PMSF, 0.5 mM benzamidine, and 1 mg/ml each of pepstatin-A, leupeptin and aprotinin). All the samples were subjected to three freeze-thaw cycles to disrupt membranes and expose enzymes before analysis. The enzymatic activity of mitochondrial respiratory chain complex I was measured at 37 °C as previously described [16]. The data were expressed as the percentage of control.

### Western blot

After various treatments, PC12 cells in 6 cm dishes were washed with ice-cold PBS for three times and lysed with a lysis buffer (50 mM Tris–HCl pH 7.5, 150 mM NaCl, 1 mM EDTA, 0.1 % SDS, 0.2 % deoxycholic acid and 1:100 protease inhibitor cocktail). The protein concentration was determined using a BCA protein assay kit. Equivalent amounts of protein (40 μg per lane) were loaded and separated by 10% SDS-PAGE gels, and transferred to polyvinylidene difluoride (PVDF) membranes. Membranes were blocked with 5% nonfat milk solution in tris-buffered saline with 0.1% Triton X-100 (TBST) for 1 h, and then incubated overnight at 4°C with the primary STIM1 antibody (1:1000; Sigma, S6072), Homer1a antibody (1:200; Santa Cruz Biotechnology, sc-8922), Bcl-2 (1:800; Cell Signaling Technology, No. 2876), Bcl-xl (1:800; Cell Signaling Technology, No. 2762), Bax (1:600; Cell Signaling Technology, No. 2772), Bad (1:800; Cell Signaling Technology, No. 9292) or β-actin antibody (1:800; Sigma, A5316) dilutions in TBST. After that the membranes were washed and incubated with secondary antibody for 1 h at room temperature. The blots were developed using chemiluminescence, and bands were detected by a GS-525 Molecular Imager with a Multi Analyst Program (Bio-Rad). An analysis software named Image J (Scion Corporation) was used to quantify the optical density of each band.

### Statistical analysis

Statistical analysis was performed using SPSS 16.0, a statistical software package. Statistical evaluation of the data was performed by one-way analysis of variance (ANOVA). A value of *p* < 0.05 was considered statistically significant.

## Results

### Effects of SOCE inhibition on MPP^+^-induced cytotoxicity

**Figure 1:**
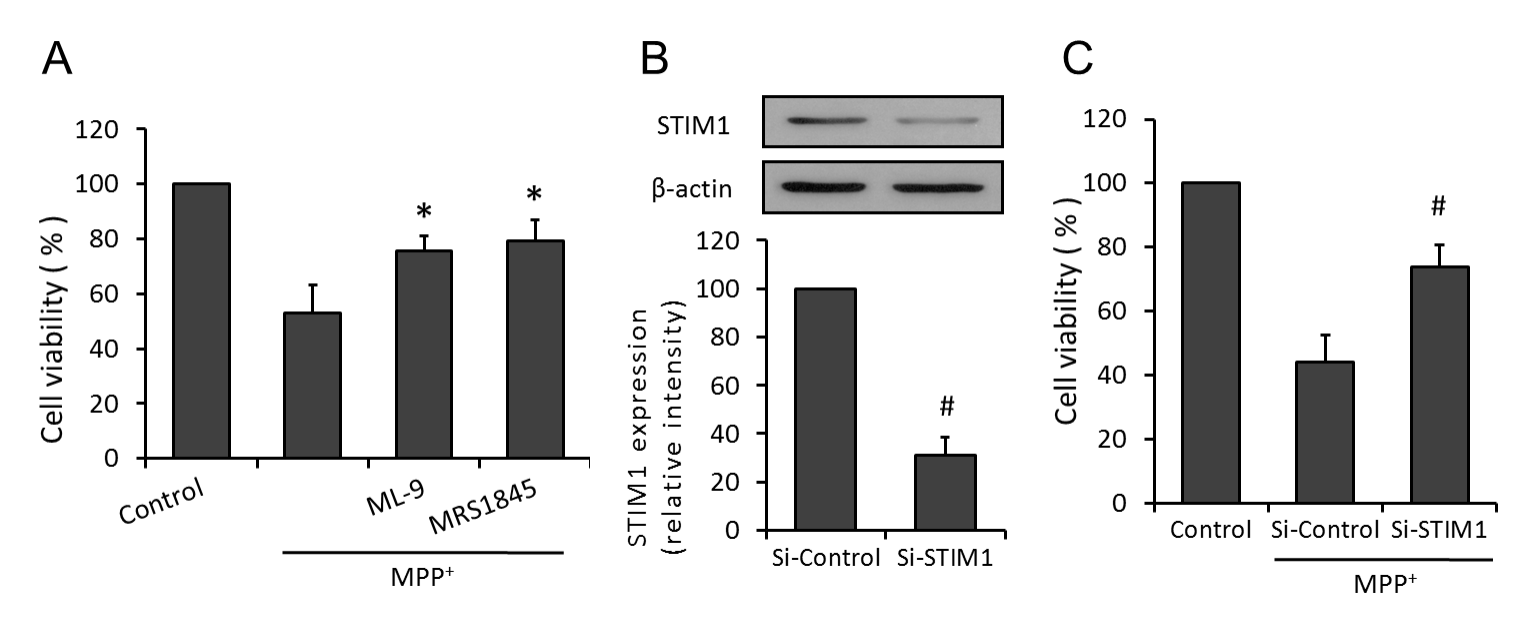
Inhibition of SOCE protects PC12 cells against MPP^+^ injury. PC12 cells were pretreated with ML-9 (50 μM) or MRS (15 μM) 30 min before MPP^+^ insult, and the cell viability was assayed 24 h later (A). After transfection with STIM1 specific siRNA (Si-STIM1) or control siRNA (Si-Control) for 72 h before MPP^+^ insult, expression of STIM1 was detected by western blot with specific anti-STIM1 antibody and cell viability was assayed 24 h later. The data were represented as means ± SD from five experiments. * *p* < 0.05 vs. MPP^+^ alone. ***#*** *p* < 0.05 vs. control siRNA.

 To investigate the potential protective effects of SOCE inhibition against MPP^+^ injury, PC12 cells were pretreated with MRS-1845 (15 μM) or ML-9 (50 μM) 30 min before insult. As shown in Figure 1A, both MRS-1845 and ML-9 obviously increased cell viability as compared with that in MPP^+^ injured PC12 cells. In order to confirm the results from pharmacological antagonists, we repeated the experiments by using specific targeted siRNA to knockdown STIM1 expression. The results showed that the cell viability of si-STIM1 transfected cells was much higher than that in si-control transfected PC12 cells (Figure 1B).

### Effects of SOCE inhibition on intracellular Ca^2+^ concentration

 In order to confirm the effects of pharmacological agents and siRNA transfection was associated with SOCE inhibition, we determined the changes of intracellular Ca^2+^ concentration by calcium imaging. Figure S1 shows the representative Ca^2+^ rise traces induced by MPP^+^ in the absence or presence of Ca^2+^ into the extracellular solution. MPP^+^ induced a transient Ca^2+^ rise in the absence of Ca^2+^, which mainly due to the ER Ca^2+^ release, and a sustained elevation in the presence of Ca^2+^, which was the results of Ca^2+^ influx through SOCE. When PC12 cells were pretreated with ML-9 or MRS1845, or transfected with Si-STIM1, the SOCE mediated Ca^2+^ influx was significantly decreased, indicating that these effects were dependent on the SOCE inhibition.

### Inhibition of SOCE attenuates MPP^+^-induced apoptosis

**Figure 2:**
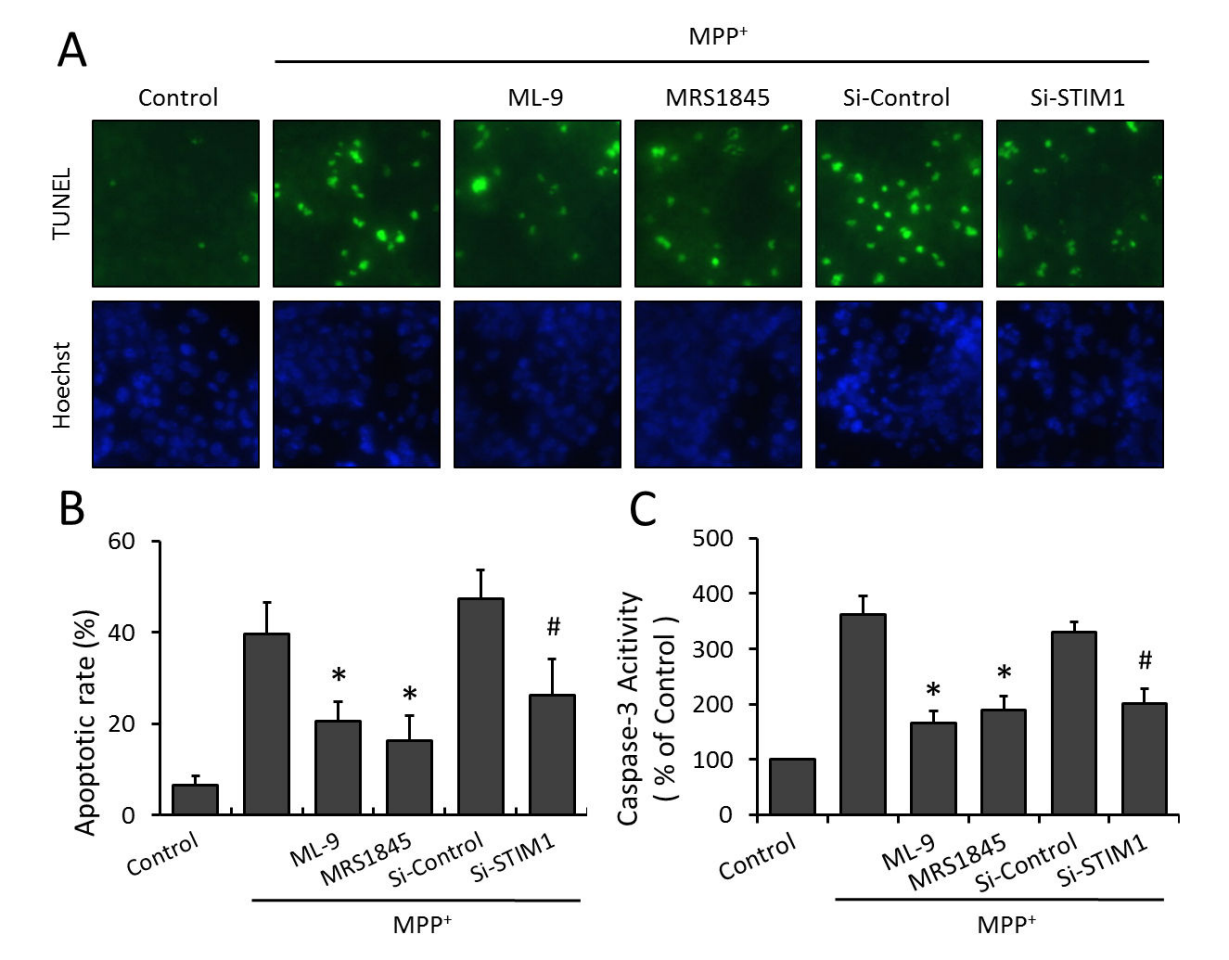
Inhibition of SOCE attenuates MPP^+^-induced apoptosis. PC12 cells were trasfected with STIM1 specific siRNA (Si-STIM1) or control siRNA (Si-Control) 72 h before MPP^+^ insult, or pretreated with ML-9 (50 μM) or MRS (15 μM) 30 min before MPP^+^ insult, and apoptotic cell death was detected by TUNEL staining (A). Apoptotic rate was represented as a percentage of the total number of cells (B). The activity of caspase-3 was measured (C). The data were represented as means ± SD from five experiments. * *p* < 0.05 vs. MPP^+^ alone. ***#*** *p* < 0.05 vs. control siRNA.

TUNEL staining was used to determine MPP^+^ induced apoptotic cell death, the main cell death type in MPP^+^ induced neuronal injury (Figure 2A). There were no obvious TUNEL-positive cells in control cells, while the number of TUNEL-positive cells significantly increased with the treatment of MPP^+^. As shown in Figure 2B, both pharmacological antagonists (ML-9 and MRS-1845) and siRNA mediated gene knockdown of STIM1 significantly attenuated MPP^+^ induced apoptotic cell death in PC12 cells. SOCE inhibition also inhibited the increased activity of caspase-3 induced by MPP^+^ insult (Figure 2C), which further confirmed the anti-apoptotic effect of SOCE inhibition in MPP^+^ injured PC12 cells.

### Inhibition of SOCE inhibits MPP^+^-induced ROS generation and lipid peroxidation

**Figure 3:**
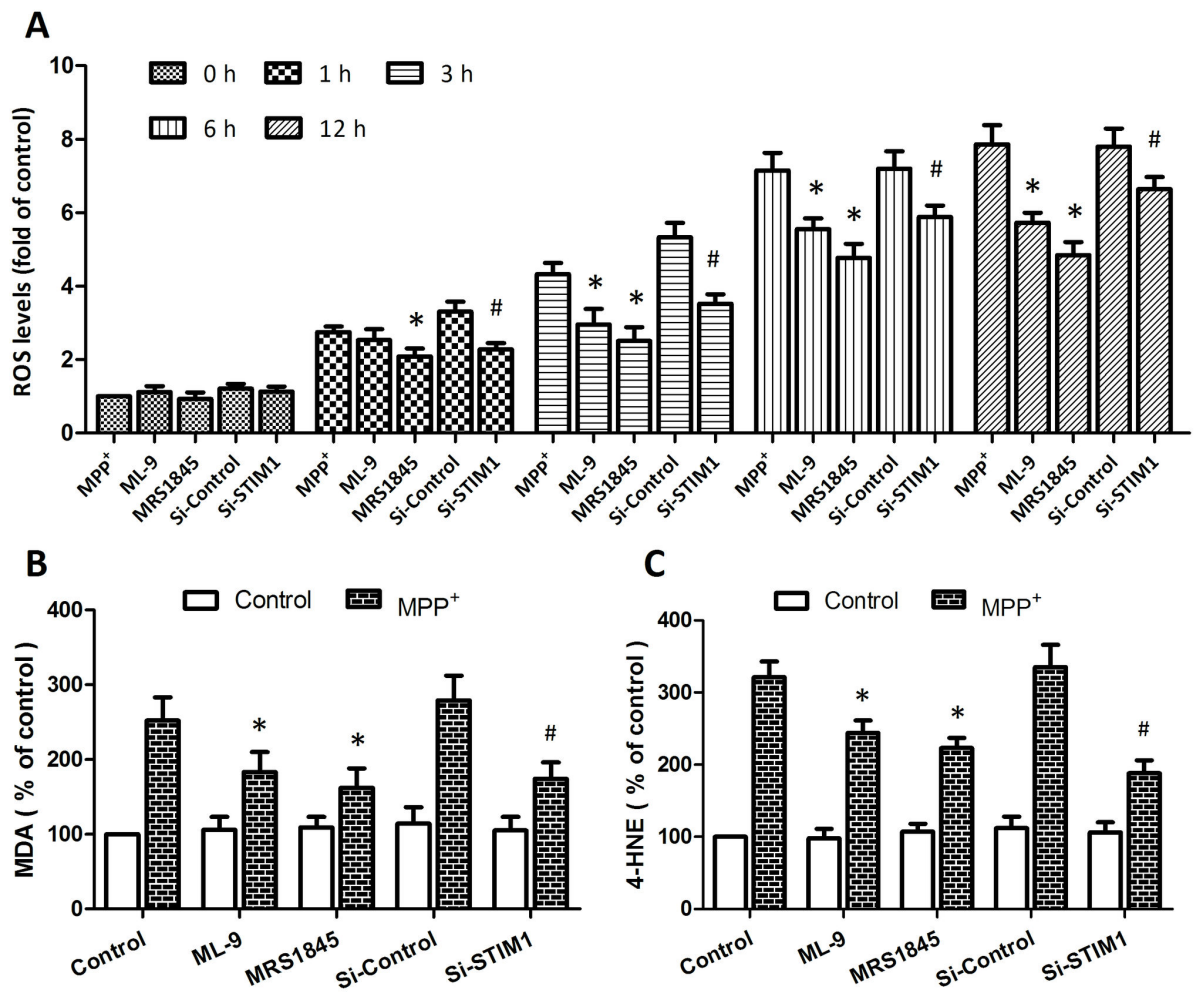
Inhibition of SOCE inhibits MPP^+^-induced ROS generation and lipid peroxidation. **PC12 cells were trasfected with STIM1 specific siRNA (Si-STIM1) or control siRNA (Si-Control) 72 h before MPP^+^ insult, or pretreated with ML-9 (50 μM) or MRS (15 μM) 30 min before MPP^+^ insult, and the intracellular ROS levels were measured at 0 h, 1 h, 3 h, 6 h, 12 h, respectively (A)**. **The levels of MDA (B) and 4-HNE (C) were assayed, respectively**. The data were represented as means ± SD from five experiments. * *p* < 0.05 vs. MPP^+^ alone. ***#*** *p* < 0.05 vs. control siRNA.

**Figure 4:**
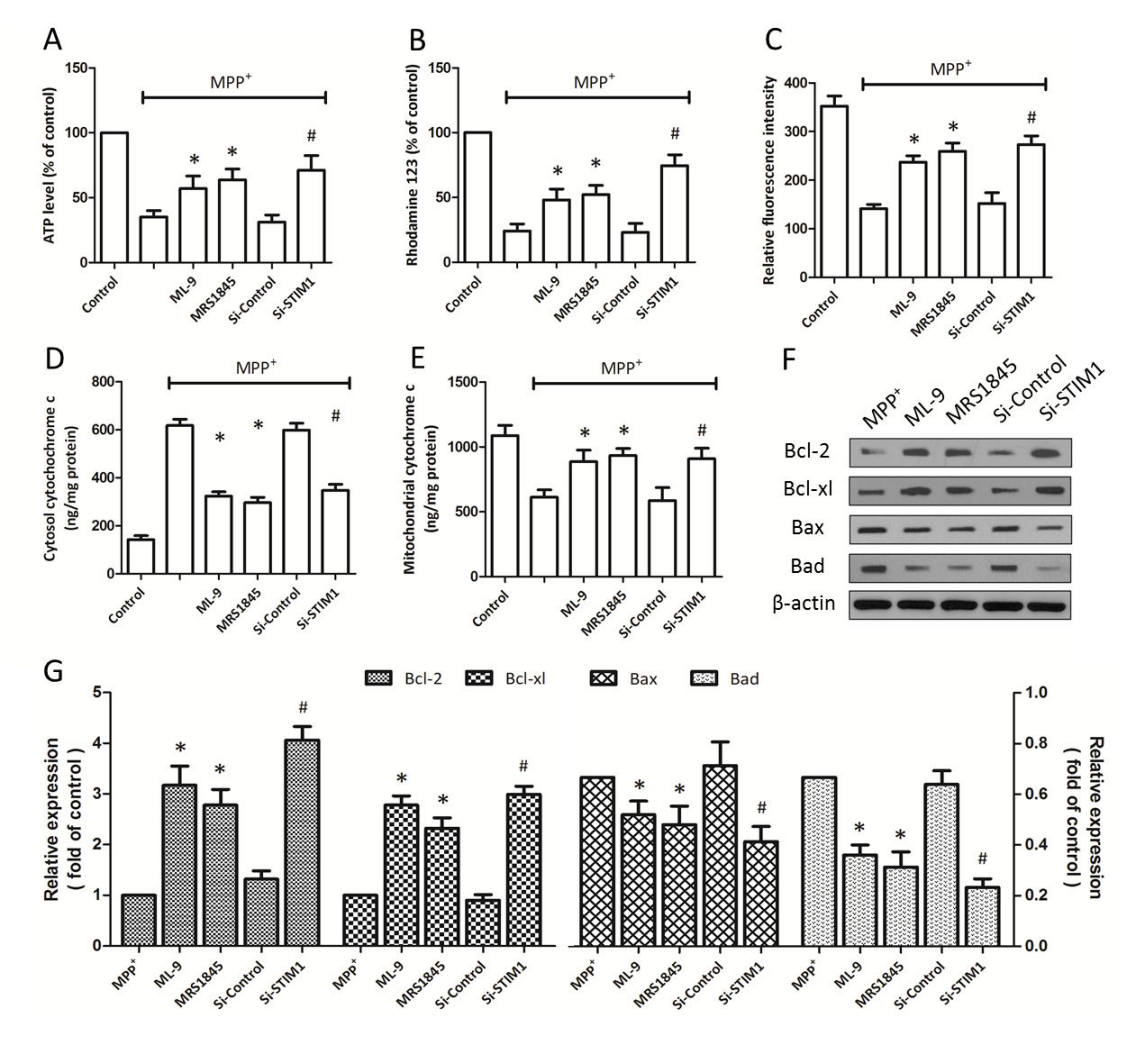
Inhibition of SOCE preserves mitochondrial function after MPP^+^ injury. PC12 cells were trasfected with STIM1 specific siRNA (Si-STIM1) or control siRNA (Si-Control) 72 h before MPP^+^ insult, or pretreated with ML-9 (50 μM) or MRS (15 μM) 30 min before MPP^+^ insult, and the ATP level (A), MMP (B) and MPTP (C) was measured, respectively. Release of cytochrome c into the cytoplasm was determined by an immunoassay kit after subcellular fraction preparation (D and E). The expression of Bcl-2, Bcl-xl, Bax and Bad were determined by western blot (F) and analyzed (G). The data were represented as means ± SD from five experiments. * *p* < 0.05 vs. MPP^+^ alone. ***#*** *p* < 0.05 vs. control siRNA.

The oxidant-sensitive probe H2DCFDA was used to detect the intracellular ROS generation. MPP^+^ injury triggered a rapid rise in intracellular ROS level that maintained to 12 h after injury, and this time dependent ROS generation was partly reversed by pretreatment of ML-9 and MRS-1845 or si-STIM1 transfection (Figure 3A). Furthermore, the levels of MDA (Figure 3B) and 4-HNE (Figure 3C) were assayed to determine the oxidative stress induced lipid peroxidation after MPP^+^ injury. Reduction of MDA and 4-HNE formation was observed in PC12 cells pretreated with ML-9 and MRS-1845 as compared to MPP^+^ insult alone, or in si-STIM1 transfected cells as compared with si-control transfected cells.

### Inhibition of SOCE preserves mitochondrial function

 To characterize the effect of SOCE inhibition on mitochondrial dysfunction after MPP^+^ injury, PC12 cells were pretreated or transfected as mentioned above. The intracellular ATP amount was measured by a colorimetric assay kit (Figure 4A), and the change of MMP was also monitored by rhodamine 123 probe (Figure 4B). Both ATP generation and MMP were reduced by MPP^+^ injury, and the reduction of ATP production and loss of MMP were increased by pretreatment of ML-9 and MRS-1845 or si-STIM1 transfection. As shown in Figure 4C, SOCE inhibition also partly reversed the MPP^+^ induced opening of MPTP, which was measured by the decreased values of relative fluorescence. 

 Furthermore, the release of cytochrome c into the cytoplasm was determined by an immunoassay kit after subcellular fraction preparation, and the results showed that SOCE inhibition decreased cytosol cytochrome c content (Figure 4D), while increased the level of mitochondrial cytochrome c (Figure 4E), indicating the inhibition of cytochrome c release. We also examined the expression of Bcl-2 family proteins by western blot (Figure 4F). As shown in Figure 4G, the MPP^+^ induced up-regulation of Bax and Bad (pro-apoptotic proteins) as well as the MPP^+^ induced down-regulation of Bcl-2 and Bcl-xl (anti-apoptotic proteins) were all partly reversed by SOCE inhibition. 

### Effects of SOCE inhibition on mitochondrial complex I activity and mitochondrial biogenesis

**Figure 5:**
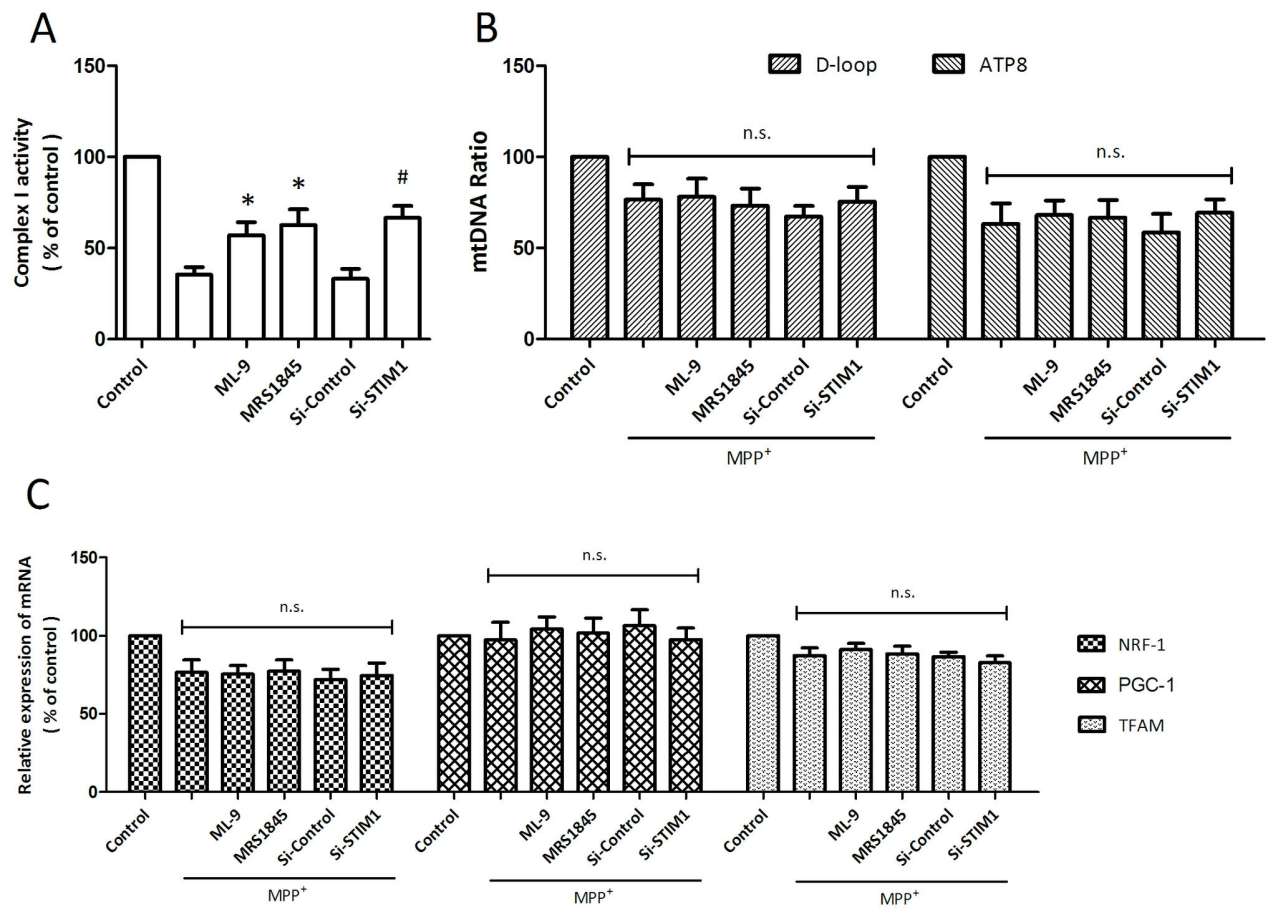
Effects of SOCE inhibition on mitochondrial complex I activity and mitochondrial biogenesis. PC12 cells were trasfected with STIM1 specific siRNA (Si-STIM1) or control siRNA (Si-Control) 72 h before MPP^+^ insult, or pretreated with ML-9 (50 μM) or MRS (15 μM) 30 min before MPP^+^ insult, and the activity of mitochondrial respiratory chain complex I was detected (A). Mitochondrial DNA content was determined by quantitative real-time PCR by comparing the mitochondrially encoded D-loop and ATP8 gene to a nuclear-encoded GAPDH gene (B), and the mRNA expression of mitochondrial biogenesis factors were measured by real-time RT-PCR (C). The data were represented as means ± SD from five experiments. * *p* < 0.05 vs. MPP^+^ alone. ***#*** *p* < 0.05 vs. control siRNA. n.s., not statistically significant.

 To investigate the potential mechanism underlying SOCE inhibition induced preservation of mitochondrial function, the activity of mitochondrial electron transport chain complex I was detected. As shown in Figure 5A, MPP^+^ injury significantly decreased the activity of complex I, which was partly reversed by SOCE inhibition. To determine whether mitochondrial biogenesis is involved in SOCE induced mitochondrial function preservation, D-loop and ATP8 were selected to detect the change of mtDNA content. As shown in Figure 5B, neither D-loop nor ATP8 replication was changed by SOCE inhibition. In addition, we also examined three transcription factors considered essential for mitochondrial biogenesis, and the results showed that the mRNA expression levels of PGC-1, NRF-1 and TFAM were not altered by SOCE inhibition, indicating a mitochondrial biogenesis independent mechanism.

### Effects of SOCE inhibition on Homer1a expression

**Figure 6:**
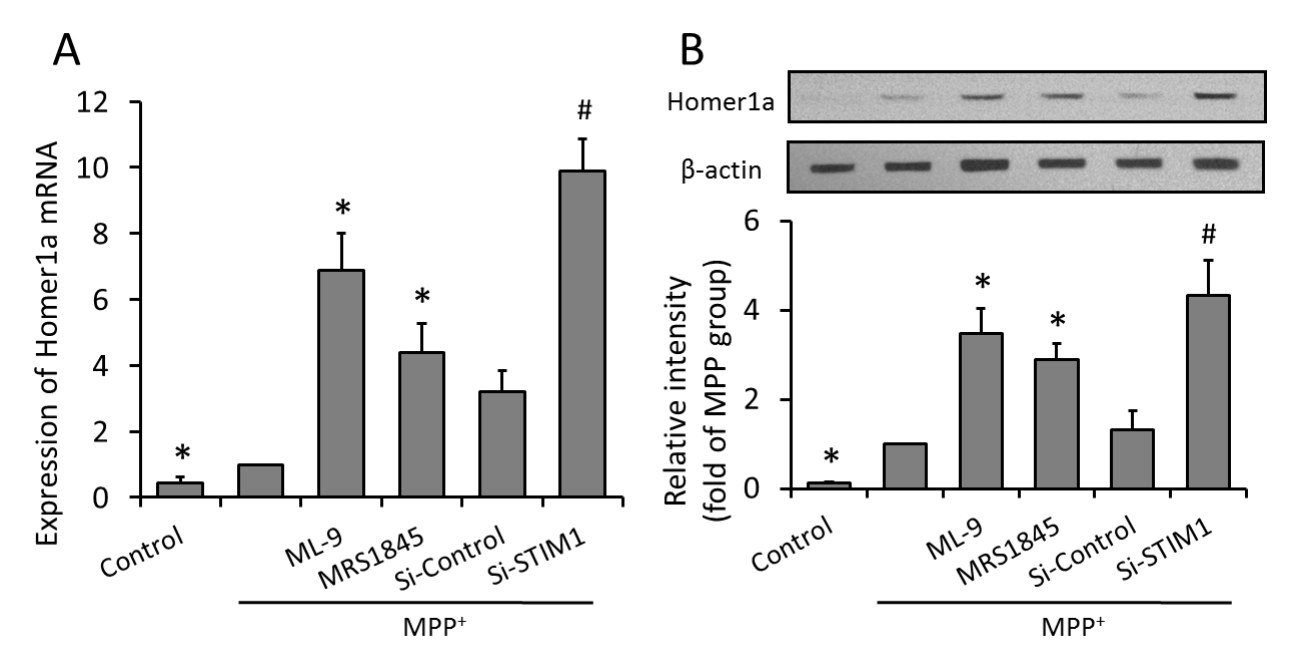
Inhibition of SOCE increases Homer1a expression. PC12 cells were trasfected with STIM1 specific siRNA (Si-STIM1) or control siRNA (Si-Control) 72 h before MPP^+^ insult, or pretreated with ML-9 (50 μM) or MRS (15 μM) 30 min before MPP^+^ insult, and the expression levels of Homer1a mRNA (A) and protein (B) were detected by Real-time RT-PCR or Western blot, respectively. The data were represented as means ± SD from five experiments. * *p* < 0.05 vs. MPP^+^ alone. ***#*** *p* < 0.05 vs. control siRNA.

 To validate the relationship between SOCE inhibition induced protection and Homer1a, we first compared the expression level of Homer1a mRNA in each group. Results obtained from real time PCR revealed that administration of ML-9 and MRS-1845 significantly increased the expression of Homer1a mRNA, and Homer1a mRNA in si-STIM1 transfected PC12 cells was much higher than that in si-control cells (Figure 6A). Additionally, results obtained from western blot revealed that SOCE inhibition also increased the expression level of Homer1a protein (Figure 6B).

### Involvement of Homer1a in SOCE inhibition induced protection

**Figure 7:**
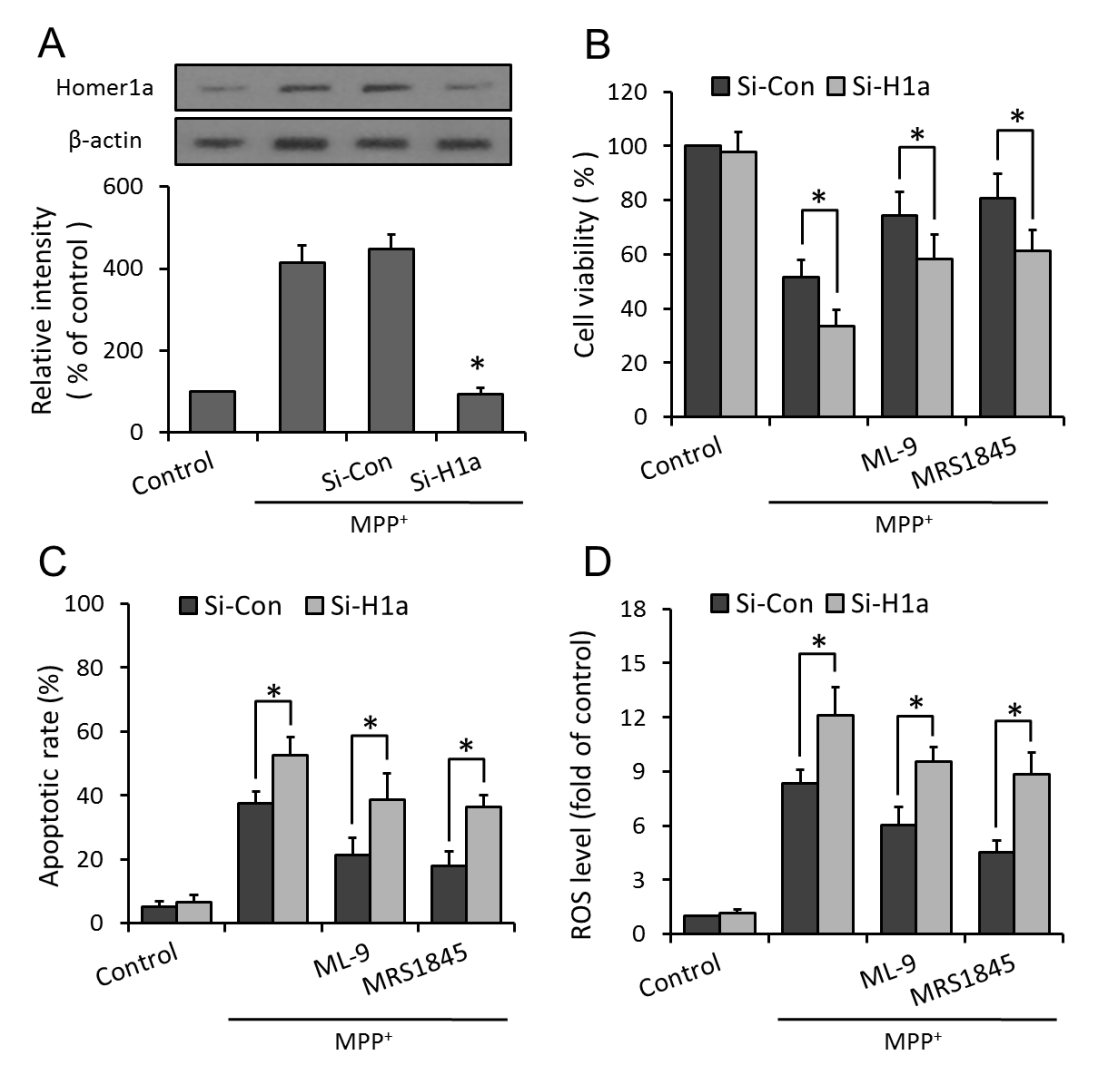
Inhibition of SOCE induced protection is dependent on Homer1a expression. PC12 cells were trasfected with Homer1a specific siRNA (Si-H1a) or control siRNA (Si-Con) for 72 h with or without pretreatment of ML-9 (50 μM) and MRS (15 μM) for 30 min before MPP^+^ insult, and the expression of Homer1a protein (A), the cell viability (B), apoptotic cell death (C) and intracellular ROS generation (D) were assayed, respectively. The data were represented as means ± SD from five experiments. *p < 0.05 vs. control siRNA.

 To further address the mechanism by which SOCE inhibition protect against MPP^+^ injury, PC12 cells were transfected with Homer1a specific siRNA or control siRNA in the presence or absence of SOCE antagonists (ML-9 and MRS-1845), and the cell viability, apoptotic cell death and intracellular ROS generation were measured, respectively. As shown in Figure 7, SOCE inhibition induced increase of cell viability and decrease of apoptosis and ROS production were all partly reversed by Homer1a knockdown, indicating that protection of SOCE inhibition was partly dependent on the increased expression of Homer1a.

## Discussion

 Changes of intracellular calcium metabolism regulate multiple cellular processes, and are considered to play important roles in a wide variety of pathological conditions, including PD. Elevations of cytoplasmic Ca^2+^ and followed activation of calcium sensitive signals could result from influx across the plasma membrane or the release of intracellular stores (most notably from the ER), and often by a combination of the two primary sources [17]. Recently, a number of publications provided evidence that these two primary sources functional linked with each other by the process of SOCE, whereby Ca^2+^ influx from extracellular space is activated in response to the depletion of intracellular calcium stores in ER [18]. After investigations for greater than 25 years, the function of SOCE was demonstrated to be not only the replenishment of intracellular Ca^2+^ stores following their discharge, but also as a regulator that can provide calcium signals to neighbouring recipients to activate downstream effectors, leading to gene expression and other appropriate responses. In the present study, we confirmed the presence of SOCE in PC12 cells, which was consistent with other in vitro studies in neurons or astrocytes [19]. We also demonstrated that functional assessment of SOCE inhibiting drugs ML-9 and MRS-1845 attenuated ROS mediated neuronal apoptosis, suggesting an involvement of SOCE in oxidative stress related apoptotic signaling pathways under MPP^+^ insult conditions.

 Although the SOCE phenomenon and its effect on calcium homeostasis were first described in 1997, the precise mechanisms and molecules that regulate this process have not been fully understood [20]. Recently, the newly discovered molecule stromal interacting molecule-1 (STIM1), a plasma membrane and ER single spanning transmembrane protein, has been demonstrated to be an essential positive regulator of SOCE at the cell based level. As a ubiquitously expressed protein in mammalian cells, STIM1 possesses dual functions: as sensor for the Ca^2+^ concentration in intracellular ER stores, and an activator for the calcium channel in the plasma membrane through translocation from ER sites into puncta that accumulated near the plasma membrane upon depletion of Ca^2+^ store [21]. Several previous studies have been done to investigate the potential involvement of SOCE mediated calcium metabolism in neurological disorders with focus on STIM1. For example, a recent study demonstrated that abnormal SOCE may participate in calcium overload of neurons in the early stages after diffuse axonal injury via enhanced STIM1 expression [22]. Moreover, an investigation on mouse embryonic fibroblasts suggested that SOCE and the sensor STIM1 are involved in the regulation of mitochondrial shape and bioenergetics and play a role in oxidative stress [23]. In contrary, some recently published papers suggested that phosphoinositide 3-kinase inhibitors wortmannin and LY294002, which were also shown to be effective in inhibiting SOCE in some cell lines, could partly reverse neuroprotective effects of some agents in MPP^+^ induced neuronal injury [24-26]. These contradictory results might be due to different downstream signaling pathways between these inhibitors, and wortmannin and LY294002 were demonstrated to effective in inhibiting several pro-survival molecules such as Akt and Bcl-2. In the present study, we found that knockdown of STIM1 expression by using specific siRNA attenuated MPP^+^ induced intracellular ROS accumulation and reduced apoptotic cell death, indicating that inhibiting STIM1 mediated SOCE associated calcium metabolism dysfunction might be a strategy for treatment of PD related neuronal injury.

 The mitochondrion is a membrane-enclosed structure found in most eukaryotic cells, and plays essential roles in signaling through mitochondrial ROS, buffering intracellular free calcium and regulating apoptotic cell death in response to neuronal injury [27]. Mitochondrial dysfunction is currently regarded as playing a central role in PD , and mitochondrial damage in MPP^+^ induced neuronal injury is consistent with intracellular ROS production, changes in MMP, opening of MPTP, release of cytochrome c and followed caspase activation [28,29], which were also confirmed in our in vitro PD models. In the present study, inhibition of SOCE significantly reversed the MPP^+^ insult induced changes of MMP, MPTP, ATP production and cytochrome c, and also preserved the activity of complex I, the most important target of MPP^+^ in neuronal cells, indicating a protective effect on mitochondrial function. Mitochondrial pathway of apoptosis is always regulated by Bcl-2 family proteins consisting of several homologous proteins including anti-apoptotic proteins (such as Bcl-2 and Bcl-xl) as well as many pro-apoptotic proteins including Bax and Bad [30-32]. Our results showed that SOCE inhibition up-regulated the expression of Bcl-2 and Bcl-xl, and down-regulated the expression of Bax and Bad. These data strongly indicate that the attenuation of mitochondrial dysfunction and regulation of Bcl-2 family proteins contribute to the neuroprotective effects of SOCE inhibition against MPP^+^ induced neuronal injury.

Homer family proteins are a group of scaffold proteins that not only assemble membrane receptors and channels at postsynaptic membranes, but also control their targeting, activity, and intracellular signaling (especially control intracellular Ca^2+^ homeostasis by regulating metabotropic glutamate receptors and voltage-gated Ca^2+^ channel activity) [13]. With a coiled-coil structure at carboxy-terminal domain, the long Homer proteins can form dimmers to functionally link their target proteins, while the short variant of Homer protein Homer 1a, which lacks the C-terminal coiled-coil structure, is considered to act as an endogenous dominant negative protein in regulating the protein complex formed by long Homer and its downstream signaling pathway [33]. A previous study has shown that Homer 1a is rapidly upregulated in spinal cord neurons after peripheral inflammation and targeted gene transfer of Homer 1a to specific spinal segments in vivo protects against chronic inflammatory pain in rats [34]. Our recent study also suggests that overexpression of Homer 1a has protective effects against H_2_O_2_-induced oxidative stress by reducing ROS accumulation and inhibiting the activation of mitochondrial apoptotic pathway [35]. Our present study suggested that blocking SOCE by using pharmacological antagonists or specific targeted siRNA increased the expression of Homer1a mRNA and protein, and knockdown of Homer1a expression by siRNA partly reversed the protective effects induced by ML-9 and MRS-1845 against MPP^+^ injury. All these results were consistent with a present investigation on primary mesencephalic cells using antagonist of canonical transient receptor potential (TRPC) channels, which have been demonstrated to be channels that mediate SOCE in neurons [36]. All the above mentioned findings, including our own in the present study, appeared presenting evidence that upregulation of endogenous Homer1a can attenuate MPP^+^ injury through activating downstream pro-survival signaling pathways, which needs to be further determined.

In conclusion, our present study demonstrated that inhibition of SOCE by using pharmacological antagonists or specific siRNA protected PC12 cells against MPP^+^ injury, and this protection was associated with the reduction of mitochondrial dysfunction mediated intracellular ROS generation and attenuation of apoptotic cell death. We also identified the molecular mechanism by which SOCE inhibition exerted protective effects through upregulation of Homer1a expression.

## Supporting Information

Figure S1 **Effects of SOCE inhibition on the intracellular Ca^2+^ concentrations.** PC12 cells were trasfected with STIM1 specific siRNA (Si-STIM1) or control siRNA (Si-Control) 72 h before MPP^+^ insult, or pretreated with ML-9 (50 μM) or MRS (15 μM) 30 min before MPP^+^ insult, and the intracellular Ca^2+^ concentration was measured by calcium imaging. Cells in control group were not treated with MPP^+^. Each trace shows the average for at least 50 cells.
(TIF)
