# Supplementary figures and images for: Inhibition of Store-Operated Calcium Entry Attenuates MPP+-Induced Oxidative Stress via Preservation of Mitochondrial Function in PC12 Cells: Involvement of Homer1a
Source: PLoS One. 2013 Dec 17;8(12):e83638. doi: 10.1371/journal.pone.0083638 (PMC3866123; doi:10.1371/journal.pone.0083638)

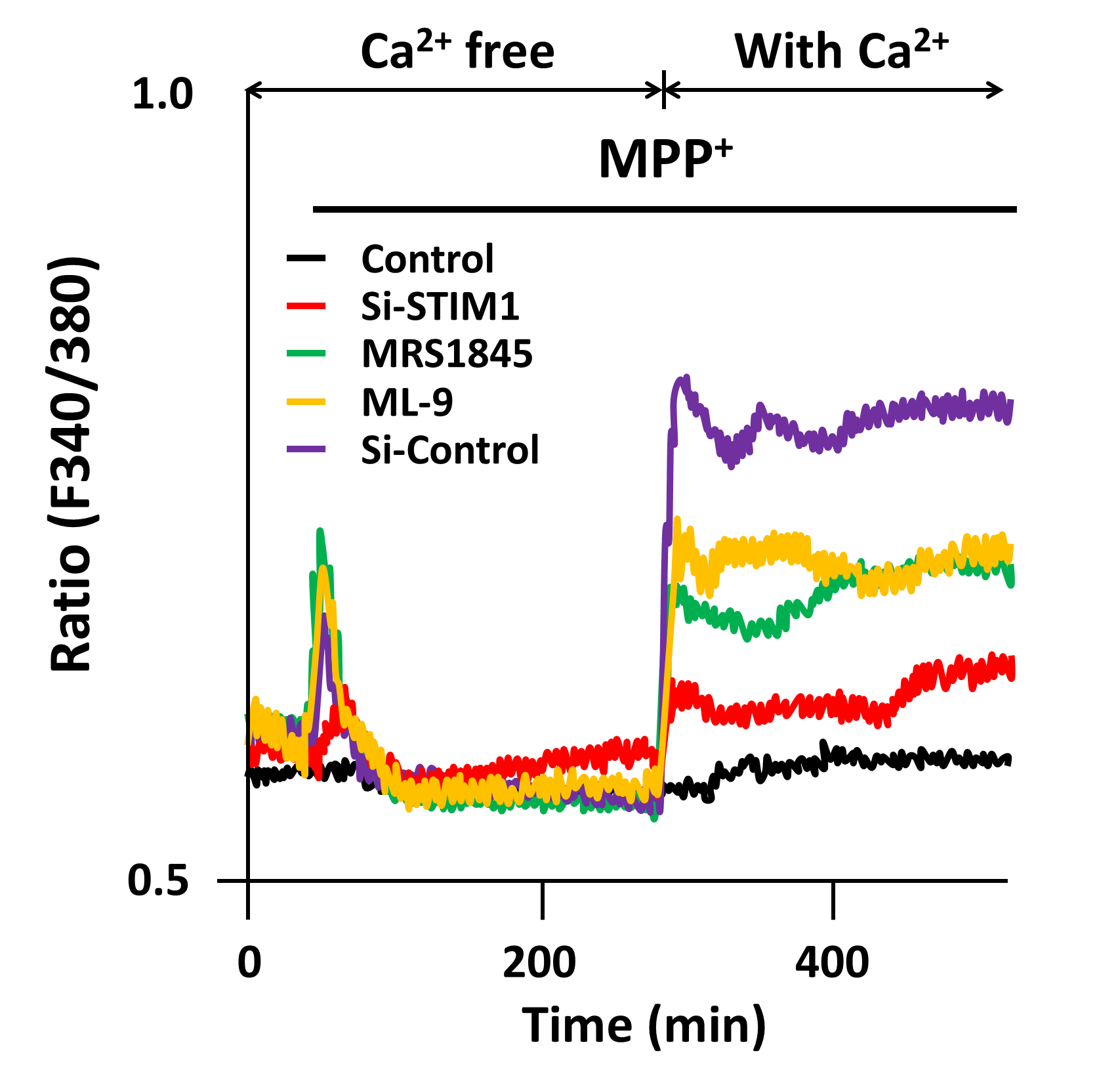

Supplement: Figure S1 — Effects of SOCE inhibition on the intracellular Ca2+ concentrations. PC12 cells were trasfected with STIM1 specific siRNA (Si-STIM1) or control siRNA (Si-Control) 72 h before MPP+ insult, or pretreated with ML-9 (50 μM) or MRS (15 μM) 30 min before MPP+ insult, and the intracellular Ca2+ concentration was measured by calcium imaging. Cells in control group were not treated with MPP+. Each trace shows the average for at least 50 cells. (TIF) [file pone.0083638.s001.tif]
